# Evaluation of bioluminescence-based assays of anti-malarial drug activity

**DOI:** 10.1186/1475-2875-12-58

**Published:** 2013-02-08

**Authors:** Sandra Hasenkamp, Adam Sidaway, Oliver Devine, Richard Roye, Paul Horrocks

**Affiliations:** 1 Institute for Science and Technology in Medicine, Keele University, Staffordshire, UK; 2 School of Life Sciences, Keele University, Staffordshire, UK; 3 School of Medicine, Keele University, Staffordshire, ST5 5BG, UK

**Keywords:** Luciferase, Sybr green I, Antimalarial drug, Relative rate of kill, Malaria

## Abstract

**Background:**

Transgenic *Plasmodium falciparum* expressing luciferase offers an attractive bioluminescence-based assay platform for the investigation of the pharmacological properties of anti-malarial drugs. Here a side-by-side comparison of bioluminescence and fluorescence-based assays, utilizing a luciferase reporter cassette that confers a strong temporal pattern of luciferase expression during the S-phase of intraerythrocytic development, is reported.

**Methods:**

Key assay parameters for a range of commercially available luminogenic substrates are determined and compared to those measured using a Malaria Sybr Green I fluorescence assay. In addition, the short-term temporal effects of anti-malarial compounds are evaluated using both bioluminescent and fluorescent assay platforms.

**Results:**

The Z’, % coefficient of variation and 50% inhibition concentrations are essentially the same for bioluminescent and fluorescent assays in transgenic parasites generated in both chloroquine-sensitive and -resistant genetic backgrounds. Bioluminescent assays, irrespective of the luminogenic agent employed, do, however, offer significantly enhanced signal-to-noise ratios. Moreover, the bioluminescent assay is more dynamic in terms of determining temporal effects immediately following drug perturbation.

**Conclusion:**

This study suggests that opportunities for bioluminescence-based assays lie not in the measurement of 50% inhibition concentrations, where the cheaper fluorescence assay performs excellently and is not restricted by the need to genetically modify the parasite clone under investigation. Instead, assays that use the dynamic response of the luciferase reporter for semi-automated screening of additional pharmacological properties, such as relative rate-of-kill and lethal dose estimation, are a more attractive development opportunity.

## Background

Following the emergence and spread of multidrug resistant *Plasmodium falciparum* and reports of artemisinin treatment failure in South-East Asia, there is an urgent need to identify new small molecule drugs for the treatment of malaria [1,2]. Towards this aim, several recent reports have described the high throughput screening (HTS) of massive chemical libraries and the identification of novel chemical scaffolds to be taken forward for development [3-5]. Prioritizing these compounds for further exploration of their pharmacokinetic and pharmacodynamic properties is essential and has been the focus of several recent developments in assay formats [6-10].

Assays of anti-malarial drug activity typically rely on either radiometric or fluorescence-based platforms. Incorporation of tritiated hypoxanthine or ethanolamine has been widely used as a biomarker for growth and offers a sensitive assay with excellent signal/noise (S/N) ratio [11]. There are, however, significant issues around the safe storage and disposal of the radiolabelled material. Fluorescence-based platforms, such as the use of the DNA intercalating agents Sybr Green I or 4^′^,6^′^-diamidino-2-phenylindole (DAPI), offer a rapid, cheap and sensitive assay but can be problematic due to the low S/N ratio resulting from high background signals [12-14]. That said, the utility of the Malaria Sybr Green I Fluorescence (MSF) assay for single-dose HTS has been readily demonstrated.

A recent innovation has been the adoption of a bioluminescence assay platform that utilizes transgenic *P*. *falciparum* expressing the luciferase reporter gene (*luc*). The utility of bioluminescence has been demonstrated in HTS of medium-sized chemical libraries, exploration of gametocidal and delayed-death drug action, and the development of a murine malaria assay in *Plasmodium berghei*[15-20]. The use of bioluminescence to assay anti-malarial drug activity in *P*. *falciparum* was first described in 2008 [17]. Here *luc* was flanked by the *Pfhsp86* 5^′^ and *Pbdhfrts* 3^′^ sequences and integrated as a short concatamer into chromosome 7. The assay provided 50% inhibition concentration (IC_50_) results comparable to those determined using ^3^H-hypoxanthine or MSF, with a subsequent report demonstrating the feasibility of scaling the use of this transgenic parasite into a 384-multiwell format [16]. Screening the Library of Pharmacologically Active Compounds (LOPAC^1280^) demonstrated the robustness of this assay format with Z’ scores >0.7 and S/N ratio of 71. In 2010, a second *luc* transgenic parasite with a *Pfhrp2* promoter was reported for the screening of three chemical libraries, a total of 12,320 compounds, reporting Z’ scores of 0.64-0.76 and S/N ratios of 147–430 [20]. The increase in S/N ratio recorded here presumably represents their choice of the Bright-Glo luminogenic substrate in their assays. A final report describes the *bxb*I integrase-mediated integration of a *Pfhrp3lucPfhrp2* cassette into chloroquine-resistant (CQR) and –sensitive (CQS) genetic backgrounds [18]. Whilst the bioluminescence assay data correlated well with that generated using ^3^H-hypoxanthine and MSF for fast-acting drugs (Z’ score of 0.71 and S/N of 51), the MSF assay appeared to provide poor resolving capacity when screening delayed-action drugs such as azithromycin (Z’ scores <0.4 and S/N of 2–3).

The generation of a transgenic parasite where *luc* is under the control of the 5^′^ and 3^′^ flanking sequences of *Pfpcna* has previously been described [21,22]. These flanking sequences have been shown to provide the regulatory elements necessary to reconstitute the absolute and temporal control of the endogenous *Pfpcna* gene on the *luc* reporter gene. *Pfpcna* encodes a processivity factor for DNA polymerase δ and, as such, acts as a biomarker for DNA replication in mature trophozoites during intraerythrocytic schizogony [23]. This transgenic parasite shows a strong 20–50,000 fold-increase in luciferase signal in mature trophozoites when compared to ring stage parasites, suggesting that the matched *Pfpcna* 5^′^ and 3^′^ flanking sequences confer a strong dynamic temporal control on *luc*[22]. Here an evaluation of the *Pfpcnaluc* transgenic parasite as a tool to explore the action of anti-malarial drugs is described along with a side-by-side comparison of this assay with MSF in both CQS and CQR genetic backgrounds. This study offers insights into the opportunities available for bioluminescence assays to explore a range of pharmacological properties of anti-malarial drugs in a format readily scalable for high throughput assays.

## Methods

### *Plasmodium falciparum* cell culture

The transgenic Dd2 *P*. *falciparum* clone expressing luciferase under the control of *Pfpcna* flanking sequences (Dd2^*luc*^) has been previously described [21]. Dd2^*luc*^ were cultured using standard continuous culture conditions (RPMI1640 medium supplemented with 37.5 mM HEPES, 10 mM D-glucose, 2 mM L-glutamine, 100 μM hypoxanthine, 25μgml^-1^ gentamycin and 8% v/v human serum) at a 2% haematocrit in an atmosphere of 1% O_2_, 3% CO_2_, and 96% N_2_. 5nM WR99210 and 2.5 μg/ml blasticidin S drug selection was applied throughout. Staging and parasitaemia were determined by light microscopy of Giemsa-stained thin blood smears. Synchronization of cultures was attained using sequential sorbitol lysis treatment [24].

### Generation of NF54^luc^ transgenic parasite

NF54^*attB*^ and pINTNeo were kindly provided by David Fidock (Columbia University, New York). *Bxb*I integrase-mediated integration of the same *Pfpcnaluc* reporter construct present in Dd2^*luc*^ was performed in NF54^*attB*^ as previously described, except that WR99210 selection was not applied as the *hdhfr* selection cassette is omitted from NF54^*attB*^[21,25]. Integration was confirmed by absence of PCR over the *cg6* locus, in which the *attB* site is located (primers P1, 5^′^ATGAACAAATACATAAGAGCGC3^′^ and P2, 5^′^TCTTTAATTTTATTTTGGTCATGC3^′^), and generation of a PCR product over the *attL* site (primers P1 and P3, 5^′^TAAGGAGAAAATACCGCATCAGG3^′^) that results from *attP × attB* recombination.

The reconstituted temporal control of *Pfpcna* expression over *luc* was shown by stage-specific northern blot and luciferase assay. NF54^*luc*^ was synchronized and samples removed at five points during asexual intraerythrocytic development. These samples represented major stages of morphological development as judged by light microscopy; early rings (0–8 hours post-infection, hpi), late rings (9-16hpi), early trophozoites (17-24hpi), late trophozoites (25-36hpi) and schizonts (37-48hpi), termed T1 to T5, respectively. Luciferase assays on T1 to T5 were carried out as described below. For stage-specific northern blots, total RNA was isolated from samples T1 to T5 and 5 μg of total RNA was size-fractionated, blotted and hybridized to α-^32^P dATP random-primer labelled probes to the *luc* and *Pfpcna* genes. Images were captured using the Cyclone Storage Phosphor screen apparatus (Packard) and analysed using OptiQuant software (Packard).

### Standard luciferase assay procedure and time course

A standard single-step lysis procedure was used throughout [22]. Forty μl samples of *P*. *falciparum* culture (typically at 2% haematocrit, HCT) were transferred to a well on a white 96-multiwell plate (Greiner, UK) and 10 μl of passive lysis buffer (Promega, UK) added and homogenized by shaking the plate. An equal volume, 50 μl, of luminogenic substrate was mixed with the lysed parasites and the bioluminescence (in relative light units, RLU) was measured for 2 sec in a Glomax Multi Detection System (Promega, UK). Four commercially available (Promega, UK) luminogenic substrates were used in this study: standard luciferase (SL) substrate, Bright-Glo^TM^, One-Glo^TM^ and Steady-Glo^TM^.

For the timecourse assay, a 2% trophozoite-stage Dd2^*luc*^ culture at 2% HCT was prepared. Five 40 μl samples were tested with each luminogenic reagent and the bioluminescent signal from each sample monitored over 100 mins immediately thereafter. Mean RLU ± standard deviation (stdev) were plotted against time. To determine the relative decay of samples, the RLU measured at each timepoint was expressed as a fraction of the initial mean RLU with the mean fraction ± stdev plotted against time.

### Dynamics of bioluminescence and fluorescence following drug treatment

A 2% early trophozoite-stage (17-24hpi) Dd2^*luc*^ culture at 2% HCT was prepared and divided into 2 ml aliquots; one for each drug/assay to be tested and a further no-drug control. At t = 0 hrs, either three 40 μl aliquots were removed to determine the initial bioluminescent signal or three 100 μl samples removed to measure the fluorescent signal (see below). The drug being tested was added at this time. At the timepoint indicated, samples of the appropriate volume were removed and the bioluminescent/fluorescent signal measured. The mean ± stdev fraction of the bioluminescent/fluorescent signal measured from the drug-treated samples was normalized against the mean bioluminescent/fluorescent signal from the no-drug control at the same timepoint.

Bioluminescent signals were measured using the protocol described above with the SL substrate. Fluorescent signals were measured using a standard MSF assay [14]. Specifically, an equal volume of MSF lysis buffer (100 μl of 20 mM Tris (pH 7.5), 5 mM EDTA, 0.008% (w/v) saponin and 0.08% (v/v) Triton X-100) containing SYBR green I (1 × final concentration, from 5000x stock supplied by Invitrogen, UK) was added to 100 μl of Dd2^*luc*^ aliquoted onto a black 96-multiwell plate (Greiner, UK). Well contents were homogenized by repeated pipetting and incubated for one hour in the dark at room temperature. The fluorescent signal, in RLU, was measured using the blue fluorescent module (excitation 490 nm: emission 510–570 nm) of a Glomax Multi Detection System (Promega, UK).

### Measuring IC_50_ using luciferase and MSF assay formats

Trophozoite-stage cultures of the transgenic line being tested (100 μl, 2% parasitaemia, 4% HCT, n = 3) were added to 96-multiwell plates containing 100 μl of pre-dosed (five-fold dilution series) complete medium. On each assay plate, six wells containing 200 μl of 2% parasitaemia cell culture (2% HCT) in the absence of drugs served as a positive control (100%), whereas the same culture mix in the presence of a 1 μM supralethal dose of artemether served as a negative growth control (0%). The outermost wells on each plate contained 200 μl of incomplete medium to minimize edge effects from evaporation during 48 hr incubation in a gassed (1% O_2_, 3% CO_2_, and 96% N_2_) chamber at 37°C.

For bioluminescent assays, RLU were measured using the SL substrate. Irrespective of the assay, the % growth was calculated as follows: 100x[μ_(S)_ − μ_(−)_/μ_(+)_ − μ_(−)_] where μ_(S)_, μ_(+)_ and μ_(−)_ represent the means for the sample in question and 100% and 0% controls, respectively. The % growth was plotted against log_10_-transformed drug concentration and the IC_50_ determined using a nonlinear regression (sigmoidal dose–response/variable slope equation) in GraphPad Prism v5.0 (GraphPad Software, Inc., San Diego, CA).

### Determination of assay quality parameters

Derivation of the statistical tests of the assay quality parameters; Z’ score, %CV_max_, %CV_min_ and signal/noise ratio was as described [26]. The Z’ score was calculated : Z^′^ = 1 − [(3σ_(+)_ + 3σ_(−)_)/ μ_(+)_ − μ_(−)_, where μ_(+)_ and σ_(+)_ are the mean and stdev of the no-drug positive control, respectively; μ_(−)_ and σ_(−)_ are the mean and stdev of a supralethal kill with 1 μM of artemether (negative control), respectively.%CV_max_ was calculated: 100x[σ_(+)_/μ_(+)_ and %CV_min_: 100x[σ_(−)_/μ_(−)_. The S/N ratio was calculated: [μ_(+)_ − μ_(−)_/σ_(−)_.

## Results

### Evaluating the parameters of the *Pfpcna*-*luc* bioluminescence assay

**Figure 1:**
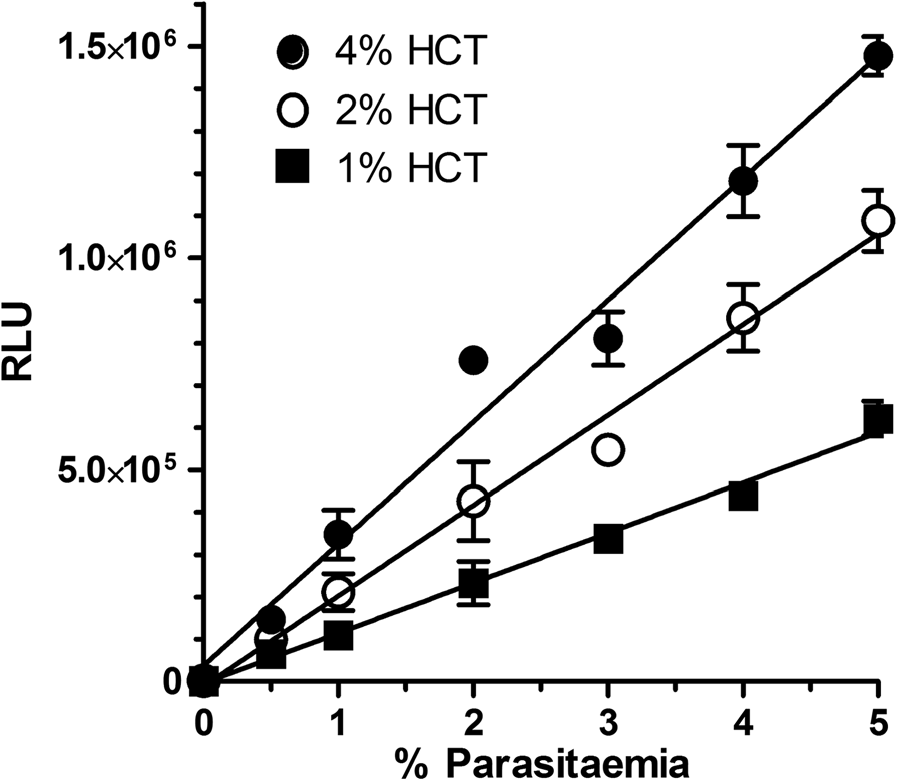
**Correlation of luciferase assay activity with haematocrit and parasitaemia. **Luciferase activity (RLU) from trophozoite stage Dd2^*luc*^ was plotted over a range of parasitaemias from cultures at three haematocrit (1, 2 and 4%, see legend). The mean RLU ± stdev (n = 5) are plotted with a linear regression line (R^2^ > 0.97 in all cases).

The generation of the *Pfpcnaluc* transgenic parasite in the Dd2 (CQR) genetic background (Dd2^*luc*^) has been previously described [21]. The haematocrit and parasitaemia starting conditions suitable for a 96-multiwell format assay using trophozoite stage Dd2^luc^ were established using a range of parasitaemia (0.5–5%) at three different haematocrit (HCT; 1,2 and 4%). The standard luciferase (SL) luminogenic substate was used to measure bioluminescence in relative light units (RLU). Correlating RLU against parasitaemia for each HCT used (Figure 1) shows a strong linear relationship (all R^2^ > 0.97) irrespective of the HCT. Increasing the parasite number through fold-increases in HCT does initially (1 to 2% HCT) result in similar fold-increases in RLU; however, the fold-increase in RLU between 2% and 4% HCT is markedly reduced due to the quenching effect of the additional haemoglobin released at these higher HCT [16,22]. Starting conditions of 2% HCT and 2% trophozoite-stage parasitaemia were selected for all future experiments. The selection of 2% parasitaemia balances the effect of kill (2% and below) and growth (assuming a 2–3 fold increase in parasitaemia) on the linear response curve. 

**Figure 2:**
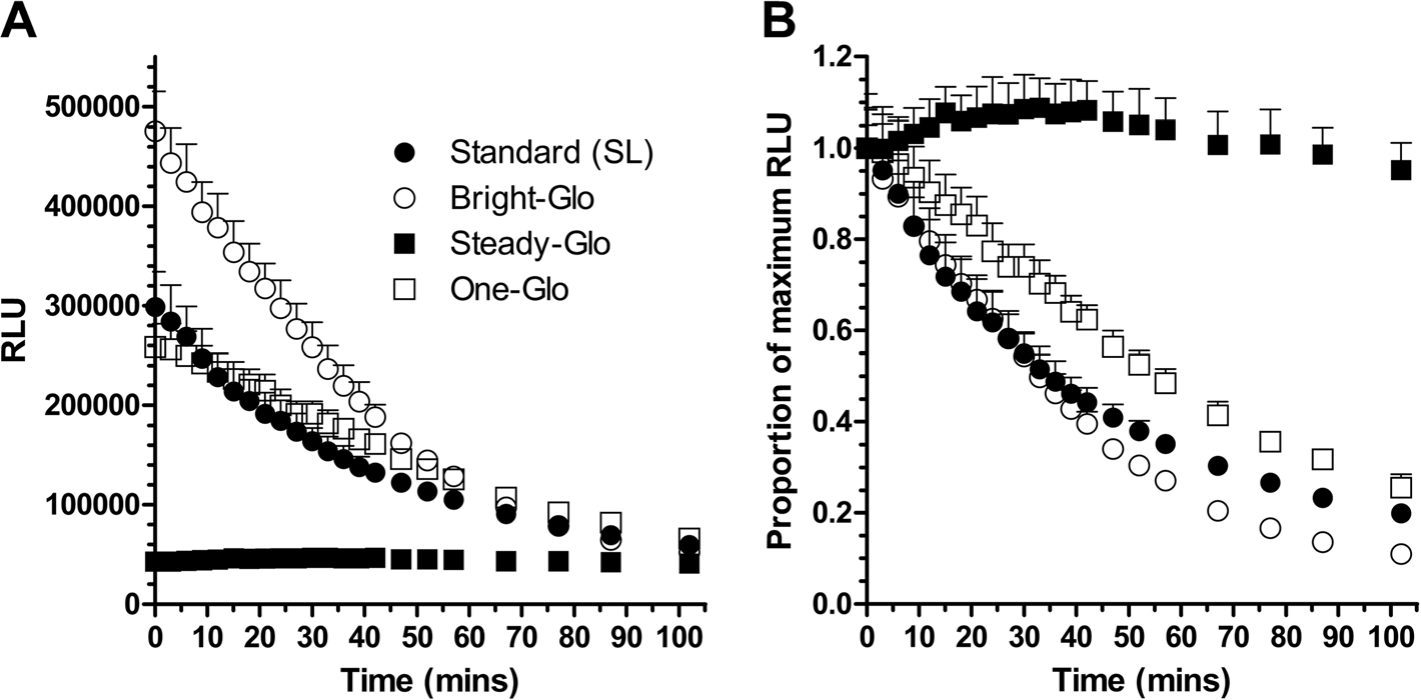
**Absolute and time-dependent decay of luminogenic substrates. **Trophozoite stage Dd2^*luc*^ (2% parasitaemia, 2% HCT) were lysed and an equal volume of the indicated (see key) luminogenic substrate added. (**A**) The decay in absolute mean value of RLU ± stdev (n = 5) for each luminogenic substrate (see key) is plotted over 100 minutes. (**B**) The relative decay for each luminogenic substrate is reported by plotting the mean RLU ± stdev as a proportion of the mean RLU at t = 0 mins.

The absolute signal intensity and time-dependent decay of RLU generated from a range of commercially available luminogenic substrates was next explored. The luminogenic substrates were added to lysed Dd2^*luc*^ and the mean RLU (n = 5) measured over 100 minutes (Figure 2A). The Bright-Glo substrate provided the highest initial absolute signal, some 60–80% higher than those for the SL and One-Glo substrates and 20-fold higher than that of Steady-Glo. Following the time-dependent decay of each luminogenic substrate, determined in terms of absolute RLU (Figure 2A) or as a fraction of initial RLU (Figure 2B), shows that the signal from the Steady-Glo substrate remains essentially unchanged over the 100 minutes assayed, whilst those of Bright-Glo, SL and One-Glo substrates decay to 10%, 22% and 28% of the original signal, respectively. Importantly, within the first five minutes, a time in which a 96-multiwell plate can be readily measured, there is a maximum loss of 10% of the initial RLU irrespective of the luminogenic substrate chosen. 

**Figure 3:**
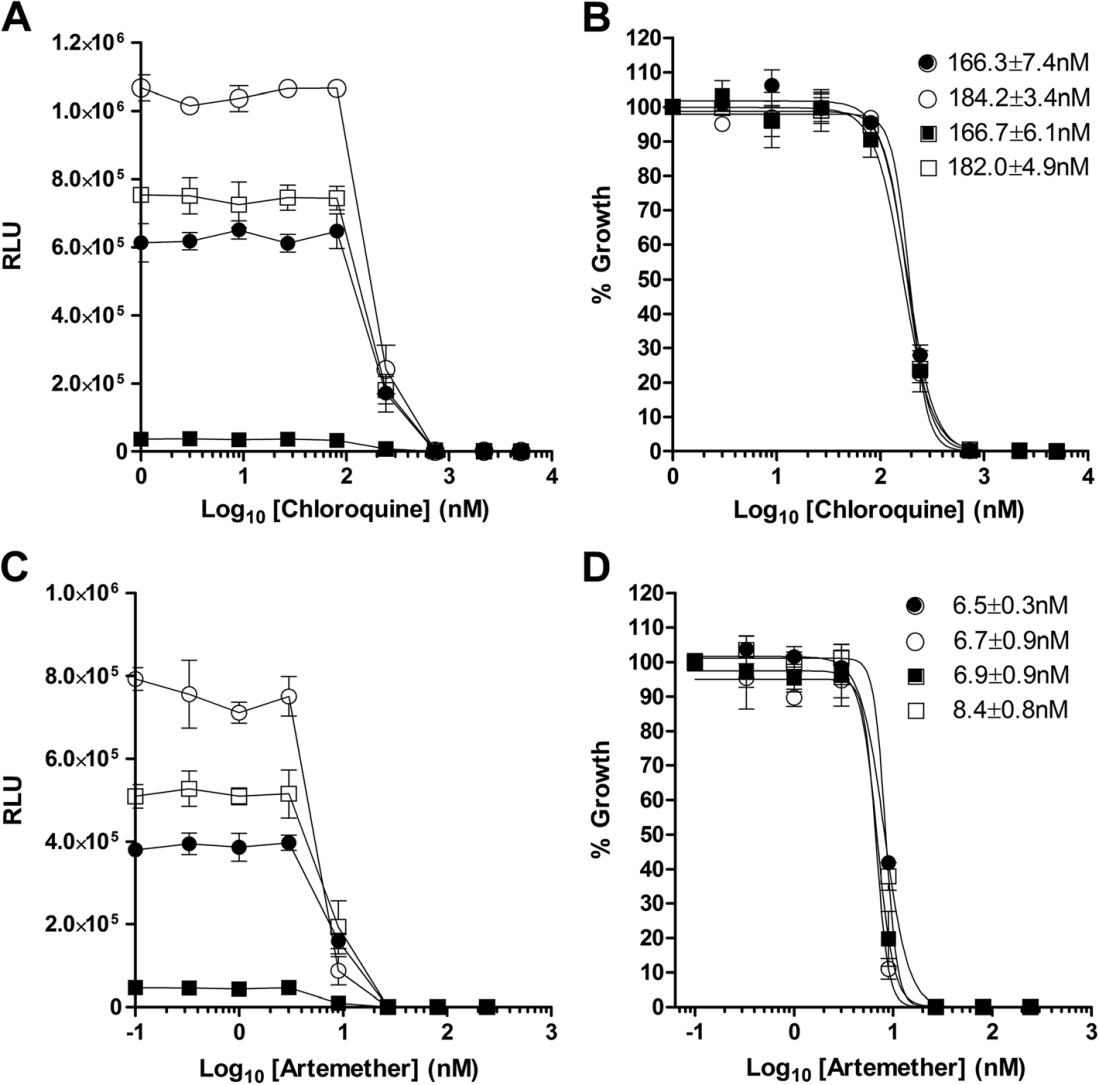
**The effect of luminogenic substrate choice on the determination of IC**_**50 **_**values. **Trophozoite stage Dd2^*luc*^ (2% parasitaemia, 2% HCT) were incubated for 48 hrs in the presence of the indicated concentration of drug prior to measurement of the absolute mean RLU ± stdev (n = 3) using (**A**) chloroquine and (**C**) artemether. Normalizing these data as a fraction of the controls (% growth) allows a dose–response curve to be plotted for each luminogenic substrate for (**B**) chloroquine and (**D**) artemether. The IC_50_ values determined for each luminogenic substrate (see key in Figure 2) are shown.

The effect of choice of luminogenic substrate for the determination of IC_50_ values was evaluated using chloroquine (CQ) and artemether (ART). Comparison of the absolute RLU (Figures 3A and 3C) over the range of drug concentrations tested shows the expected relative relationship. Normalizing the RLU as a fraction of the untreated control for each luminogenic substrate generates overlapping non-linear sigmoidal dose–response curves that provide essentially identical IC_50_ values irrespective of the substrate chosen (Figures 3B and 3D). Ten samples of Dd2^*luc*^ exposed for 48 hrs to either no drug (100% growth) or a supralethal dose of ART (1 μM, 0% growth) allow the Z’ score, S/N ratio and coefficient of variation (%CV) assay parameters to be determined for each luminogenic substrate in a 96-multiwell format (Table 1). For comparison, the same assay parameters were determined using the MSF assay (Table 1). These data indicate that whilst the Z’ scores are essentially the same for all the assay formats tested, and all formats would be readily suitable for HTS, there were significant differences in the S/N ratios. Critically, luminogenic substrate provides S/N ratios between 469 and 4042 fold higher than those of the fluorescence-based MSF assay. As all the luminogenic substrates share a similar and very low background signal (50–80 RLU), the fold increase in S/N ratio correlates with the initial absolute RLU generated by each substrate. The low variation in the maximal signal (100% growth, %CV_max_) across all the luminogenic substrates demonstrates the robust performance of the bioluminescence assays in generating consistent data. The higher %CV_min_ provided by the bioluminescence assays is a direct result of variation of the very low background signal (50–80 RLU). 

**Table 1:** Comparison of assay parameters from bioluminescence and fluorescence-based assays

**Assay reagent**	**Z’score**	**Signal/noise ratio**	**%CV**_**max**_	**%CV**_**min**_
Bright-Glo	0.74–0.84	15667–17746	5.2–6.7	14.8–16.2
SL substrate^1^	0.73–0.81	12853–13609	8.0–8.9	15.5–17.0
One-Glo	0.77–0.86	8936–10030	4.7–6.3	14.3–19.5
Steady-Glo	0.83–0.86	2063–2344	5.6–7.1	17.2–18.6
MSF	0.72–0.79	4.30–4.39	4.0–6.6	2.8–5.8

^1^Standard luciferase (SL).

### Bioluminescence is a more dynamic reporter of drug action

The fluorescence and bioluminescence assays of Dd2^*luc*^ both monitor DNA replication. MSF directly monitors DNA content and bioluminescence measures the S-phase-linked induction of expression of DNA replication proteins. However, the DNA biomarker is much more stable than the luciferase biomarker, and each is subject to distinct cellular regulation processes. To explore the effect of the stability of these different biomarkers, a lethal drug perturbation was induced and the immediate dynamic responses of the bioluminescence and fluorescent assays followed. For the bioluminescence assays, the SL substrate was selected for use throughout, its choice reflecting a balance of signal intensity, stability and relative cost.

Dd2^luc^ was exposed to supralethal doses of drug perturbation; kill was induced using 1 μM of the RNA polymerase inhibitor Actinomycin D (ActD) or the ribosome translational elongation inhibitor cyclohexamide (CHX). Over 8 hrs, samples were harvested and the bioluminescent/fluorescent signal measured. In each case, the RLU measured were expressed as a fraction of the mean RLU from an untreated control harvested at the same timepoint to compensate for the normal temporal programme of *luc* expression over the course of the experiment.

**Figure 4:**
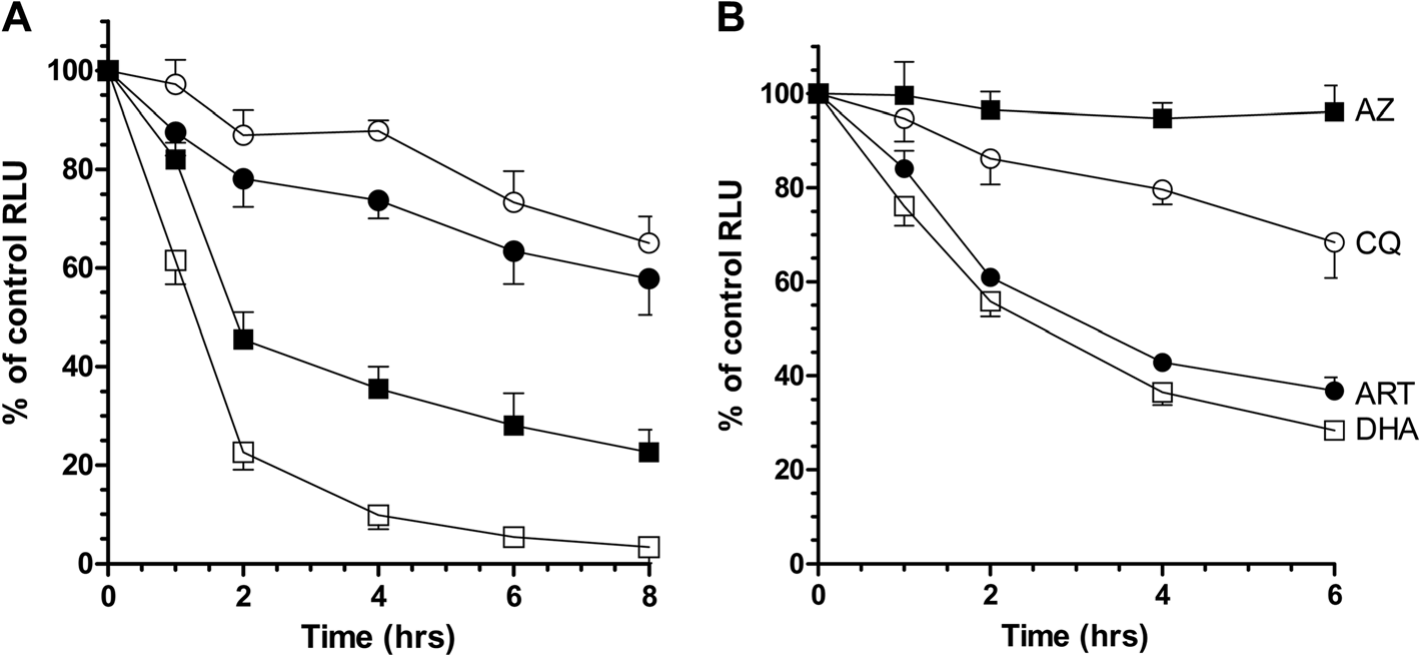
**Exploring the immediate dynamic response of bioluminescence and fluorescence assays of drug activity. **(**A**) Plot of the time-dependent changes in relative RLU (bioluminescent and fluorescence when compared to untreated control) measured from trophozoite stage Dd2^l*uc*^ (2% parasitaemia, 2% HCT) exposed to a supralethal 1 μM dose of actinomycin D (filled symbol) or cyclohexamide (open symbol). The fraction of mean RLU ± stdev (n = 3) at each timepoint are shown for a luciferase assay (square) or MSF assay (circle). (**B**) Plot of the time-dependent changes in mean fraction of luciferase RLU ± stdev (n = 3) exposed to 3xIC_50_ doses of the indicated anti-malarial drug; artemether (ART), azithromycin (AZ), dihydroartemisinin (DHA) and chloroquine (CQ).

Kill induced by either drug results in decreasing bioluminescent/fluorescent signal over the 8 hours assayed (Figure 4A). Whereas the bioluminescent signal decreased to between 3 and 22% of the untreated control (square symbols in Figure 4A), the fluorescent signal only decreased to between 57 and 65% of the untreated control over the same period (circles in Figure 4A). Interestingly, the fluorescent signal initially decreased more on ActD treatment, whereas the bioluminescent signal was most affected by CHX. These data suggest that the action of ActD appears to initially reduce Sybr Green I binding, presumably through low-affinity binding to a component of double-stranded RNA, with the inhibition of *de novo* DNA synthesis by CHX slightly delayed. The residual 65% of fluorescence even after 8 hours of supralethal drug treatment presumably reflects the stability of the nucleic acid biomarker even after cell death. By contrast, supralethal doses of CHX rapidly block the *de novo* synthesis of luciferase and the induced kill leads to a rapid decline in bioluminescence. Over the 8 hours of treatment, the bioluminescent signal decreases to 3% of the untreated control, with the time-response curve indicating that the luciferase protein has a half-life of approximately 1.5 hours in *P*. *falciparum*. The action of ActD appears slightly delayed, presumably as *luc* transcript is available for translation prior to cell death. 

Based on the initial dynamic response of the bioluminescence assay to ActD and CHX, the effect of anti-malarial drugs was similarly tested. Four drugs were selected; dihydroartemisinin (DHA, 20nM), artemether (ART, 25nM), chloroquine (CQ, 500nM) and azithromycin (AZ, 6 μM) with the concentration of each drug representing the 3xIC_50_ dose (see below). The time-response curves generated provide relative rate-of-kill data that follows the known relative action of these drugs (Figure 4B) [10]. The artemisinins ART and DHA provide the fastest rate of kill. CQ represents an intermediate between the artemisinins and AZ, a drug that shows a delayed-death phenotype. The killing-effect of AZ would not be expected to be exerted within the 8 hour time period used here, instead requiring the parasite to complete at least one full cycle of intraerythrocytic development [18].

### Side by side comparison of bioluminescence v. fluorescence determination of IC_50_

**Figure 5:**
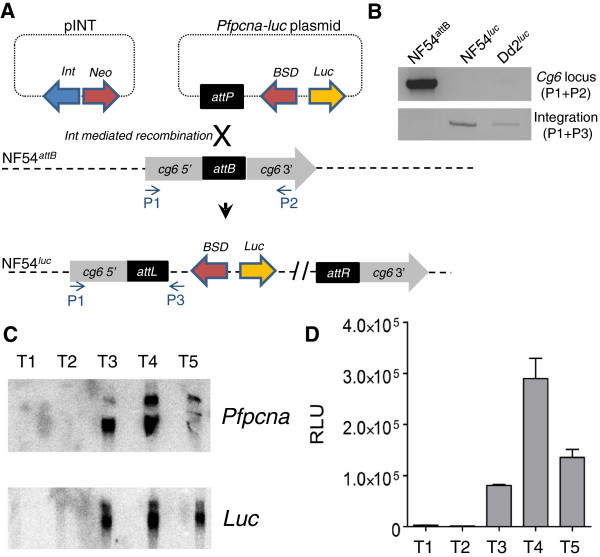
**Generation of the NF54**^***luc ***^**transgenic line. **(**A**) Schematic representing the *bxb*I-integrase (*Int*) mediated integration of the *Pfpcna*-*luc* plasmid into the *cg6*-*attB* locus in NF54^*attB*^. *BSD* and *Neo* represent the drug selection markers for blasticidin S and neomycin, respectively. The position and orientation of oligonucleotide primers (P1 to P3) used to confirm integration are shown. (**B**) PCR monitoring of integration of *Pfpcna*-*luc* plasmid. The top panel shows results of PCR across the *cg6*-*attB* locus (primers P1 and P2), and the lower panel, PCR across the *attL* junction (primers P1 and P3) resulting from successful integration in NF54^*luc*^. Dd2^*luc*^ is included as control. In the case of Dd2^*luc*^, no product is expected across the *cg6*-*attB* locus as this is disrupted with the *hdhfr* drug selection cassette. (**C**) Stage-specific northern blot using probes to *Pfpcna* and *luc* transcripts. The expected sizes of the predominant transcripts of 1.8Kb and 2.6Kb, respectively, were found. T1 to T5 represent timepoints of sampling from early rings, late rings, early trophozoites, late trophozoites and schizonts, respectively. (**D**) Time-course of luciferase sampling of NF54^*luc*^ (2% parasitaemia, 2% HCT) over T1–T5. The bars represent mean RLU ± stdev (n = 5) using the standard luciferase substrate.

As luciferase expression in Dd2^*luc*^ is temporally linked to DNA replication, this parasite offers the most appropriate bioluminescence assay for a side by side comparison with the DNA intercalating action of Sybr Green I in the MSF assay. To extend the comparison of IC_50_ derived from bioluminescence and fluorescence assays using the *Pfpcnaluc* cassette, this cassette was also introduced into the CQS genetic background provided by NF54. The *Pfpcnaluc* cassette was introduced into NF54^*attB*^ by *bxb*I-mediated integration (Figure 5A) as confirmed by PCR analysis (Figure 5B). To demonstrate that the same temporal control of *Pfpcna* was reconstituted over the *luc* gene in NF54^*attB*^, stage-specific northern blots and luciferase assays were performed (Figures 5C and 5D). Taking five timepoints (T1 to T5) that represent major morphological stages during asexual intraerythrocytic development (early ring, late ring, early trophozoite, late trophozoite and schizont, respectively), the expected patterns of temporal steady-state transcript levels and luciferase activity were demonstrated, with the highest levels for both coinciding with S-phase in late trophozoites (sample T4) [22]. 

Using the luciferase transgenic lines Dd2^*luc*^ and NF54^*luc*^, the IC_50_s for eight anti-malarial drugs, representing a range of modes of action, were determined using both bioluminescence and MSF assays (Table 2). The parental lines Dd2^*attB*^ and NF54^*attB*^ were included using the MSF assay alone as a control to explore any effect on IC_50_ resulting from the genetic modification with the *Pfpcna*-*luc* cassette. Irrespective of the assay format, the IC_50_s determined for these drugs were essentially the same. The differences in IC_50_ for CQ and quinine (QN) between Dd2^*luc*^ and NF54^*luc*^ reflect known differences in their resistance profiles for these quinolone drugs. Determining the mean IC_50_ ratio for Dd2^*luc*^/NF54^*luc*^ for CQ gave values of 8.57 and 8.91 for the bioluminescent and MSF assays, respectively. Similarly, for QN, the ratios were almost identical; 2.38 and 2.28, and reflect typical values for these drugs in CQR/CQS parasites. The IC_50_ values derived using the MSF assay for the docking parasite lines Dd2^*attB*^ and NF54^*attB*^ were similarly unchanged from the MSF assays of the related *luc* transgenic lines. 

**Table 2:** **Comparison of IC**_**50 **_**derived from bioluminescence and fluorescence-based assays**

**Drug**^**1**^	**MSF**		**Luc**	**MSF**		**Luc**
	**Dd2**^**attB**^	**Dd2**^**luc**^	**Dd2**^**attB**^	**NF54**^**attB**^	**NF54**^**luc**^	**NF54**^**luc**^
Artemether	12.0 ± 0.5	11.3 ± 0.3	8.4 ± 0.8	12.1 ± 1.3	13.1 ± 0.9	12.2 ± 0.9
Atovaquone	1.9 ± 0.4	1.3 ± 0.6	1.7 ± 0.1	0.7 ± 0.6	0.9 ± 0.2	2.9 ± 0.3
Azithromycin	>1580	>1580	>1580	>1580	>1580	>1580
Cloroquine	22.6 ± 2.5	26.4 ± 1.0	18.6 ± 2.5	26.7 ± 1.0	13.2 ± 2.1	21.0 ± 1.3
Dihydroartemisinin	133.0 ± 7.8	125.6 ± 2.4	166.3 ± 7.4	7.9 ± 0.7	14.1 ± 2.2	19.4 ± 1.8
Mefloquine	4.4 ± 0.5	7.8 ± 1.2	6.0 ± 0.4	9.7 ± 1.4	7.1 ± 1.2	5.6 ± 0.3
Quinine	173.5 ± 13.0	157.3 ± 20.0	115.4 ± 9.1	62.6 ± 5.7	69.0 ± 3.2	48.5 ± 4.0
WR99210	nd^2^	nd	nd	nd	0.55 ± 0.13	0.48 ± 0.21

^1^IC_50_ values (n.3 independent measurements in the triplicate) reported as mean ± standard deviation in nM, ^2^not done.

## Discussion

The utility of bioluminescence in single-dose HTS of small- to medium-sized chemical libraries has been readily demonstrated, providing evidence for the luciferase reporter assay as a robust and reliable platform for anti-malarial drug discovery [16,18,20]. An evaluation of a range of commercially-available luminogenic substrates suitable for scale-up of this assay platform are evaluated here. This evaluations shows that key assay parameters such as the Z’-score and % coefficient of variation are essentially the same irrespective of the luminogenic substrate selected. Whilst Steady-Glo appears to offer a significant advantage due to its relative signal stability, particularly when considering use of 384 or even 1536-microwell plates, this is simply ameliorated through the use of microinjection devices that deliver substrate immediately prior to detection of the bioluminescent signal. Thus, the standard luciferase substrate, the only commercial luminogenic substrate investigated here that is not co-formulated with lysis reagent (and thus less prone to foaming on microinjection) would perhaps represent the best choice for scale-up. Critically, however, bioluminescence would appear to offer few advantages over the current choice of Sybr-Green I fluorescence assay for single-dose HTS. The advantages offered by the higher S/N ratio and very low background of bioluminescence, whilst extremely helpful for ready analysis of the data, are significantly outweighed by the increased cost of luminogenic substrate and the restriction of study to only those *P*. *falciparum* lines that are genetically modified to express luciferase.

The opportunities for bioluminescence would thus appear to lie elsewhere. One such area is the exploration of stage-specific activity of anti-malarial drug action. Currently, in addition to the late trophozoite-specific expression of luciferase reported here, *P*. *falciparum* transgenic lines that express peak luciferase levels in ring, schizont and different gametocyte stages exist [15,17,20]. The advantages in very high S/N ratio reported here, however, are not as apparent in the studies that describe these other transgenic parasites. For example, whilst this study reports S/N ratios between 15667 and 17746 using the Bright-Glo substrate, the same substrate only provided S/N ratios up to 430 in a transgenic parasite where the *luc* gene is under the control of a *Pfhrp2* promoter [20]. These findings may result from technical issues associated with choice of luminometer or perhaps, more likely, from a need to better understand the role of *luc* flanking sequences. Using matched *Pfpcna* 5^′^ and 3^′^ flanking sequences, a complete reconstitution of the absolute and temporal control of the endogenous gene over the *luc* reporter can be done [21]. This same observation is similarly true using a second gene, PF0660w, which is expressed in trophozoite-stage parasites (Hasenkamp *et al*. submitted). These findings would suggest that use of matched flanking sequences from genes subject to high levels of temporally-linked expression may offer the opportunity to develop a library of transgenic parasites suitable for the screening of stage-specific anti-malarial drug activity.

The high rate of luciferase turnover, however, may offer the most attractive opportunity for assay development. Inducing kill using supralethal doses of the translation inhibitor CHX indicates that luciferase has an approximate 1.5 hr half-life in *P*. *falciparum*. This high rate of reporter turnover, accompanied by high quantum yield of light from strong matched regulatory sequences, may be exploited to facilitate the *in vitro* investigation of the immediate dynamics of drug treatment. Rate-of-kill is a critical determinant of anti-malarial drug action and ideally this should be as fast as possible [1]. Rapid rate-of-kill is a specific stated attribute in the target product profile for a single-exposure radical cure drug, with the intention of rapidly reducing the parasite burden, and thus clinical symptoms of disease ( http://www.mmv.org). Rate-of-kill is typically first characterized during phase IIa clinical trials but an *in vitro* assay of rate-of-kill has been recently described [10]. As this recrudescence-based *in vitro* assay of rate-of-kill assay requires some 21–28 days post-drug selection to develop the data, the utility of a more rapid *in vitro* assay to explore the rate-of-kill of anti-malarial drugs is potentially of interest in establishing priorities for anti-malarial drug development programmes.

## Conclusion

This study provides proof-of-principle data supporting the application of bioluminescent assays to determine the relative rate-of-kill of four anti-malarial drugs. The relative rate-of-kill as judged from bioluminescent assays is the same as that previously reported from *in vivo* PRR and *in vitro* recrudescence rate-of-kill assays [10]. Critically, drugs that exhibit the same mode of action show similar relative rates-of-kill in this novel assay format. Importantly, these assays were performed in a 96-multiwell plate format over a six-hour window which would suggest some potential for development into a rapid *in vitro* rate-of-kill assay more amenable for scale up.

## Competing interests

The authors declare no competing interests.

## Authors’ contributions

SH carried out the molecular and drug assay studies and drafted the manuscript. AS and OD carried out the initial evaluation of bioluminescence assays and comparison to MSF assays. RR measured the IC50 values in the CQR and CQS transgenic lines. PH designed the study, carried out the rate-of-kill experiments and prepared the final manuscript. All authors read and approved the final manuscript.
